# Prevalence and impact of acute renal impairment on COVID-19: a systematic review and meta-analysis

**DOI:** 10.1186/s13054-020-03065-4

**Published:** 2020-06-18

**Authors:** Xianghong Yang, Yiyang Jin, Ranran Li, Zhongheng Zhang, Renhua Sun, Dechang Chen

**Affiliations:** 1 Department of Critical Care Medicine, Zhejiang Provincial People’s Hospital, Hangzhou Medical College, Hangzhou, 310014 Zhejiang People’s Republic of China; 2 College of Letters & Science, University of California, Berkeley, Berkeley, CA 94720 USA; 3 Department of Critical Care Medicine, Ruijin Hospital, Shanghai Jiao Tong University School of Medicine, Shanghai, 200025 People’s Republic of China; 4 Department of Emergency Medicine, Sir Run Run Shaw Hospital, Zhejiang University School of Medicine, Hangzhou, 310016 Zhejiang People’s Republic of China; 5 Department of Critical Care Medicine, Ruijin North Hospital, Shanghai Jiao Tong University School of Medicine, Shanghai, 201800 People’s Republic of China

**Keywords:** 2019-nCoV, SARS-CoV-2, Renal impairment, Acute kidney injury, Continuous renal replacement therapy, Meta-analysis

## Abstract

**Background:**

The aim of this study is to assess the prevalence of abnormal urine analysis and kidney dysfunction in COVID-19 patients and to determine the association of acute kidney injury (AKI) with the severity and prognosis of COVID-19 patients.

**Methods:**

The electronic database of Embase and PubMed were searched for relevant studies. A meta-analysis of eligible studies that reported the prevalence of abnormal urine analysis and kidney dysfunction in COVID-19 was performed. The incidences of AKI were compared between severe versus non-severe patients and survivors versus non-survivors.

**Results:**

A total of 24 studies involving 4963 confirmed COVID-19 patients were included. The proportions of patients with elevation of sCr and BUN levels were 9.6% (95% CI 5.7–13.5%) and 13.7% (95% CI 5.5–21.9%), respectively. Of all patients, 57.2% (95% CI 40.6–73.8%) had proteinuria, 38.8% (95% CI 26.3–51.3%) had proteinuria +, and 10.6% (95% CI 7.9–13.3%) had proteinuria ++ or +++. The overall incidence of AKI in all COVID-19 patients was 4.5% (95% CI 3.0–6.0%), while the incidence of AKI was 1.3% (95% CI 0.2–2.4%), 2.8% (95% CI 1.4–4.2%), and 36.4% (95% CI 14.6–58.3%) in mild or moderate cases, severe cases, and critical cases, respectively. Meanwhile, the incidence of AKI was 52.9%(95% CI 34.5–71.4%), 0.7% (95% CI − 0.3–1.8%) in non-survivors and survivors, respectively. Continuous renal replacement therapy (CRRT) was required in 5.6% (95% CI 2.6–8.6%) severe patients, 0.1% (95% CI − 0.1–0.2%) non-severe patients and 15.6% (95% CI 10.8–20.5%) non-survivors and 0.4% (95% CI − 0.2–1.0%) survivors, respectively.

**Conclusion:**

The incidence of abnormal urine analysis and kidney dysfunction in COVID-19 was high and AKI is closely associated with the severity and prognosis of COVID-19 patients. Therefore, it is important to increase awareness of kidney dysfunction in COVID-19 patients.

## Introduction

Previous publications have shown that AKI developed in 5 to 15% cases and carried a high mortality rate (60 to 90%) in SARS and MERS-CoV infections [1]. Recent studies have reported that acute renal impairment also occurs in COVID-19 patients. Cheng et al. have shown that among 710 consecutively hospitalized patients with COVID-19, 44% had proteinuria and hematuria, and 26.7% had hematuria on admission [2]. The incidence of elevated sCr and BUN was 15.5% and 14.1%, respectively [3]. It has also been reported that the incidence rate of AKI in COVID-19 patients ranged from 0.5 to 29% dependent on the different severity of the illness. The AKI incidence was 0.1 to 2% for mild cases, 3–3.2% for severe cases, and up to 8.3–29% for critically ill patients that need to be admitted into the ICU [4–13].

In order to fully understand the prevalence and the clinical characteristics of kidney impairment in COVID-19 patients, we have provided in this article a systematic review not only to evaluate the prevalence of acute renal impairment, but also to assess the risk of AKI in severely ill patients and non-survivors compared to non-severely ill patients and survivors, respectively. The findings in this article will help the clinicians to increase awareness of kidney impairment in COVID-19 patients.

## Methods

### Data source, search strategy, and exclusion criteria

**Fig. 1:**
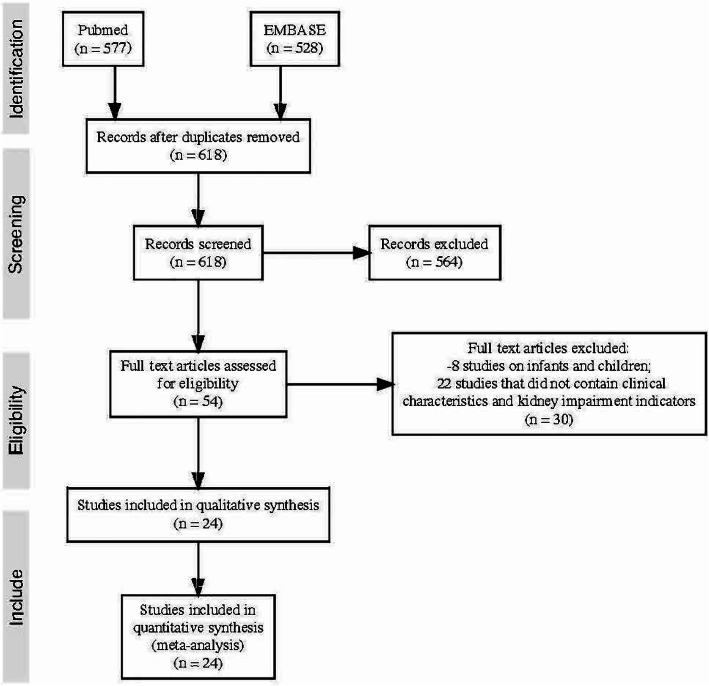
Flow diagram of the selection process to identify the studies included in the meta-analysis

We conducted a systematic search on PubMed and Embase from December 2019 to May 2020 and have used terms “COVID-19” and “novel coronavirus” in combination with terms including “clinical characteristics,” “kidney injury,” and “renal impairment” as keywords for literature search. The exclusion criteria included [1] studies that duplicate [2], studies on sample size smaller than 10 [3], studies that do not provide useful clinical characteristics or kidney impairment indicators [4], case reports, reviews or editorials [5], only children cases and family-based studies, and [6] studies written in Chinese (for the fear of data duplication). The literature search is shown step by step with a flow chart (Fig. 1). Two investigators worked independently to decide which studies should be included, and the disagreement was resolved by a third investigator.

### Data extraction

After literature retrieval, we extracted the following parameters from 24 articles: sCr, BUN, elevation of sCr, elevation of BUN, incidence of AKI, proteinuria, proteinuria +, proteinuria ++/+++, the incidence of AKI in severely (including severe cases and critical cases) and non-severely (including mild and moderate cases) ill patients, the incidence of AKI in survivors and non-survivors, application of CRRT in severely ill and non-severely ill patients, and application of CRRT in survivors and non-survivors. The characteristics of these studies are shown in Table 1.

**Table 1:**
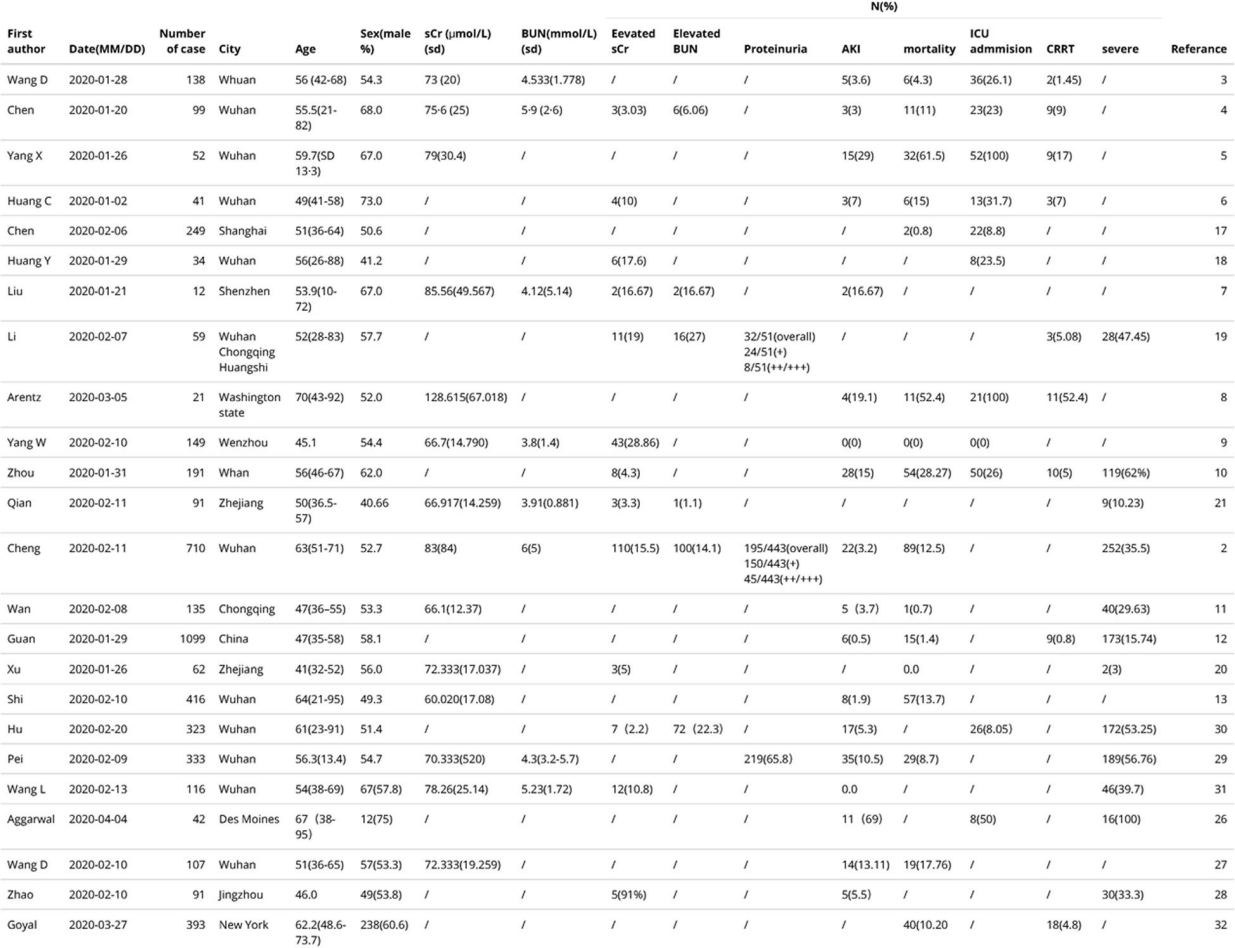
Number, age, sex, and kidney impairment indicators, AKI incidence and CRRT application on COVID-19 patients of the 24 studies included

### Data analysis

All analyses and plots were performed and made with the R version 3.6.3. For studies that did not provide mean and standard deviation but provided median (m) and upper (q3) and lower quartiles (q1) instead, the mean was estimated by (q1 + m + q3)/3, and the standard deviation was estimated by (q3 − q1)/1.35 [14]. Forest plots were made to show the estimated mean and 95% confidence interval of kidney indicators or prevalence and the corresponding 95% CI of kidney impairment, respectively. The log risk ratios (log RR and 95% CI) were calculated to illustrate the relative risk of severe or non-survival patients to show AKI or to be supported with CRRT compared with non-severe or survival patients, respectively. The level of heterogeneity is defined based on the calculated *I*^2^: an *I*^2^ smaller than 25% represents insignificant heterogeneity, an *I*^2^ between 25% and 50% represents low heterogeneity, an *I*^2^ between 50% and 75% represents moderate heterogeneity, and an *I*^2^ larger than 75% represents high heterogeneity [15]. A fixed effect model (inverse variance) was used to pool the data if *I*^2^ < 50%, and a random effect model (DerSimonian-Laird) was used if *I*^2^ > 50% [16]. The threshold of statistical significance in this paper was set to be 0.05.

## Results

We initially identified 306 articles based on the search results, and 102 articles were recognized to be duplicates. After removing the duplicates, the team members have carefully reviewed the titles, the abstracts, and the tables of the remaining 29 articles, and identified 24 articles [4–13, 17–28] that met our selection criteria and contained the data we need to investigate. These 24 articles were all published in 2020, and a total number of 4963 patients were included. The sample patient size of the groups ranged from 12 to 1099, and most of the studies recruited are based on patients in China, with three exceptions that focused on patients in the USA (Table 1).

**Fig. 2:**
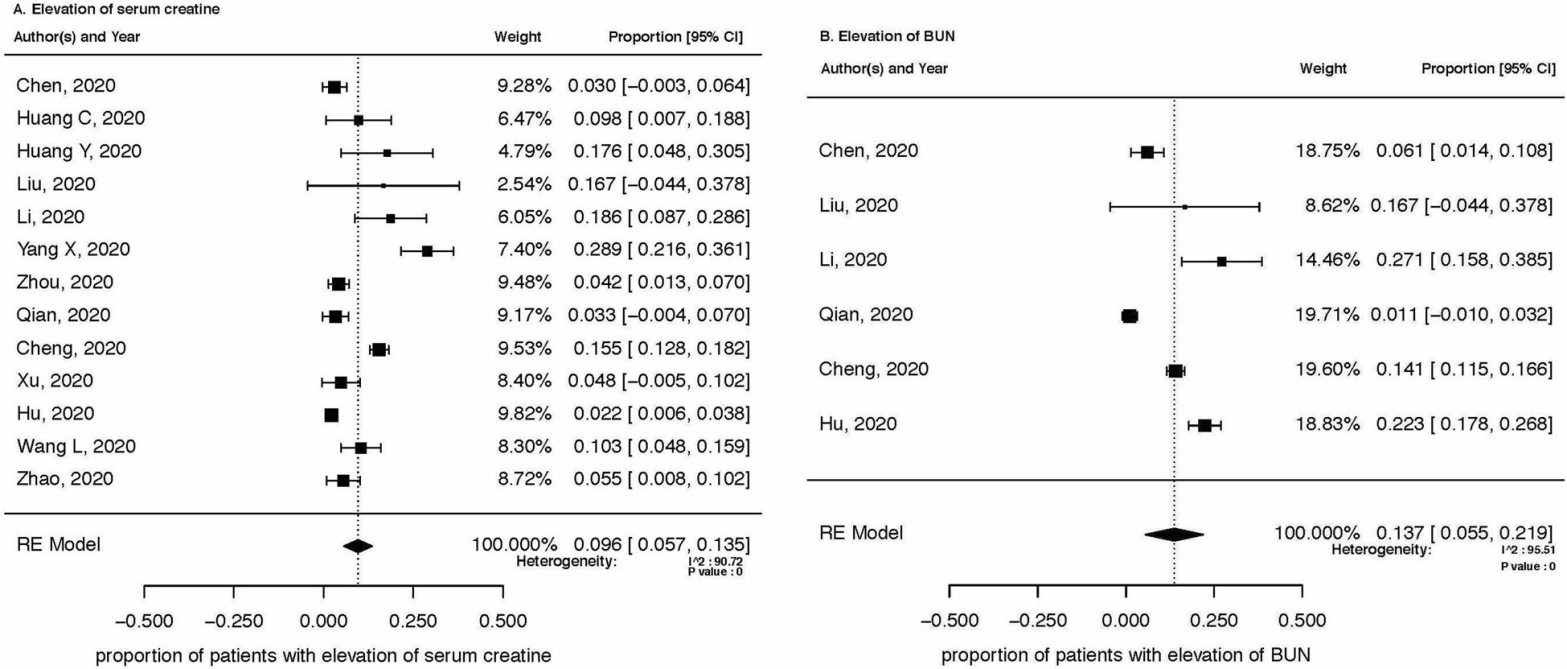
Meta-analysis of the proportion of COVID-19 patients with elevation of serum creatinine and BUN. Heterogeneity is defined based on the calculated *I*^2^ index, and random effect models are used to calculate the weights. The forest plots represent proportion of patients with elevation of serum creatinine and BUN (**a**, **b**)

**Fig. 3:**
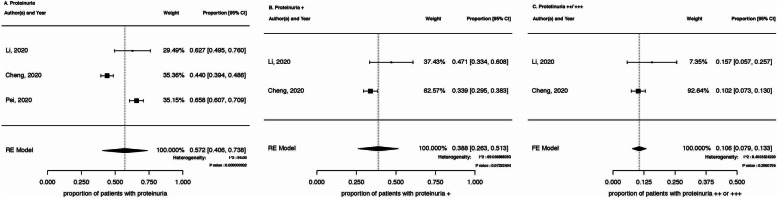
Meta-analysis of incidence of proteinuria in COVID 19 patients. The forest plots represent the average incidence of proteinuria (**a**), proteinuria + (**b**), and proteinuria ++/+++ (**c**) in COVID-19 patients. Heterogeneity is defined based on the calculated *I*^2^ index. Based on the heterogeneity, random effect models are used to calculate the weights in **a** and **b**, and the fixed effect model is used to calculate weights in **c**

Meta-analyses of these studies have shown that 9.6% (95% CI 5.7–13.5%) and 13.7% (95% CI 5.5–21.9%) of the patients showed an elevation in the sCr level and BUN level, respectively (Fig. 2). In addition, we have also conducted meta-analysis on the incidence of proteinuria of COVID-19 patients. Based on the three studies that contained data about proteinuria, 57.2% (95% CI 40.6–73.8%) of all patients had proteinuria, 38.8% (95% CI 26.3–51.3%) of all patients had proteinuria +, and 10.6% (95% CI 7.9–13.3%) of all patients had proteinuria ++ or +++ (Fig. 3). Heterogeneity was included in the above-mentioned kidney impairment indicators except for the proportion of patients with proteinuria ++ or +++, as the *I*^2^ indexes were between 69.1 and 96.9%, which were all above 50%. The *I*^2^ index of 8.40% (*P* value = 0.30) of the proteinuria ++ or +++ indicated that homogeneity was included.

**Fig. 4:**
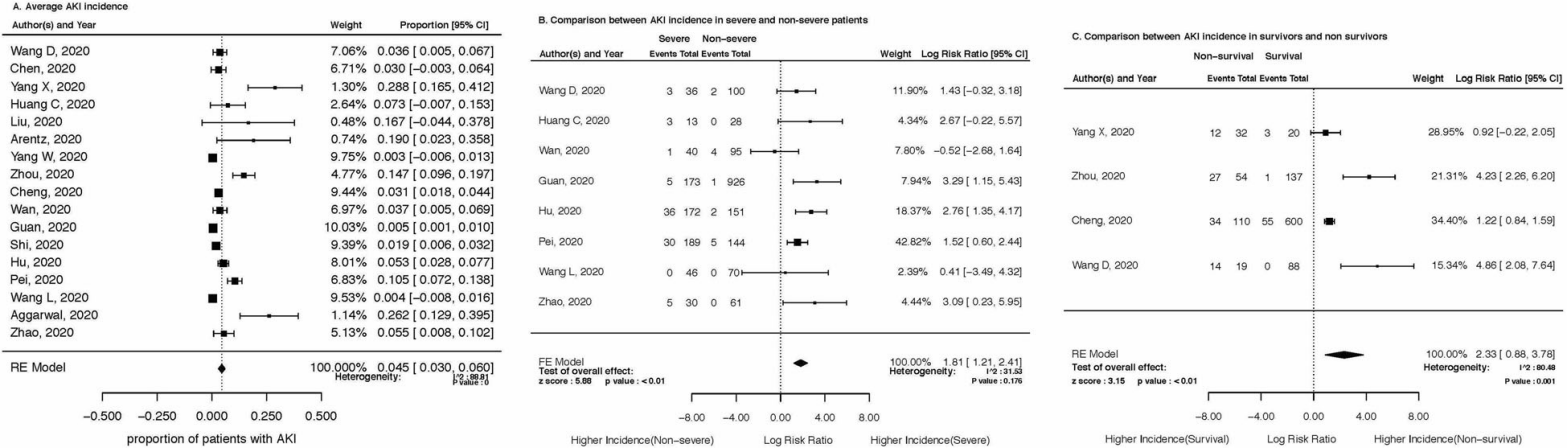
**a** Forest plot of average AKI incidence in all COVID-19 patients. **b** Forest plots of AKI log risk ratio between severe and non-severe patients. **c** Forest plots of AKI log risk ratio between survival and non-survival cases. Heterogeneity is defined based on the calculated *I*^2^ index, and random effect models are used to calculate the weights

**Fig. 5:**
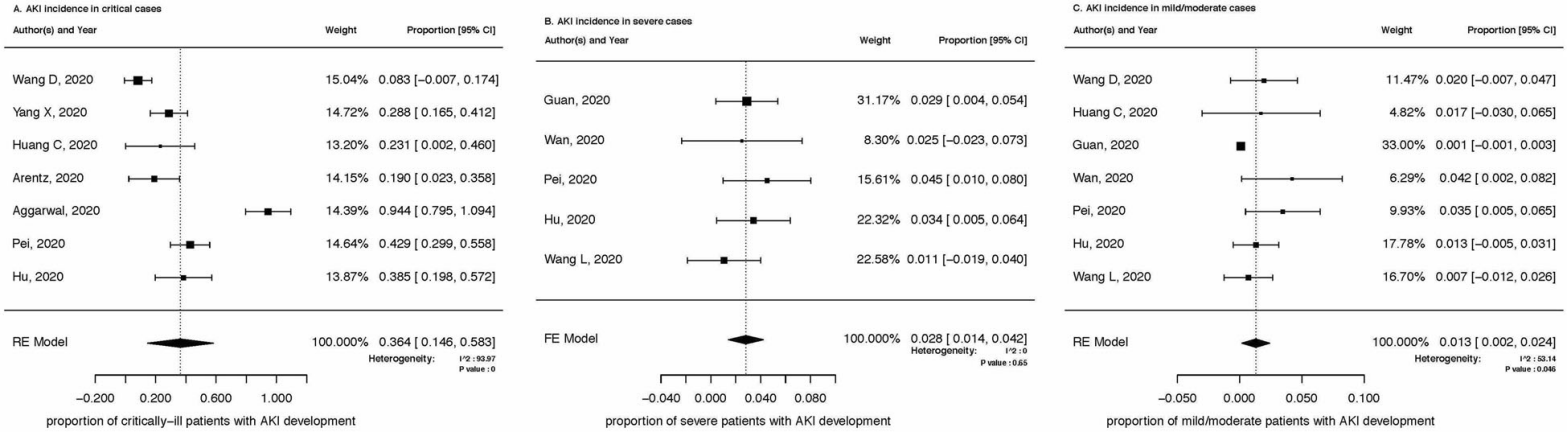
**a** Forest plot of average AKI incidence in critical COVID-19 cases. **b** Forest plot of average AKI incidence in severe COVID-19 cases. **c** Forest plot of average AKI incidence in mild/moderate COVID-19 cases. Heterogeneity is defined based on the *I*^2^ index calculated. A random effect model is used to pool the average AKI incidence in critical patients, and fixed effect models are used to pool the data of AKI incidence in severe and mild/moderate cases

The meta-analysis has shown that 4.5% (95% CI 3.0–6.0%) of COVID-19 patients had AKI (Fig. 4). The incidence of AKI was 1.3% (95% CI 0.2–2.4%), 2.8% (95% CI 1.4–4.2%), and 36.4% (95% CI 14.6–58.3%) in mild or moderate cases, severe cases, and critical cases, respectively (Fig. 5). The log risk ratio for severe patients to develop AKI compared with non-severe patients was 1.81 (95% CI 1.21–2.41, *Z* = 5.88, *P* < 0.01, Fig. 4). The meta-analysis also showed that the incidence of AKI was 52.9%(95% CI 34.5–71.4%) vs 0.7%(95% CI − 0.3–1.8%) in non-survivors and survivors, respectively (Supplementary Figure 1). The log risk ratio to develop AKI for non-survivors compared to the survivors was 2.33 (95% CI 0.88–3.78, *Z* = 3.15, *P* < 0.01, Fig. 4).

**Fig. 6:**
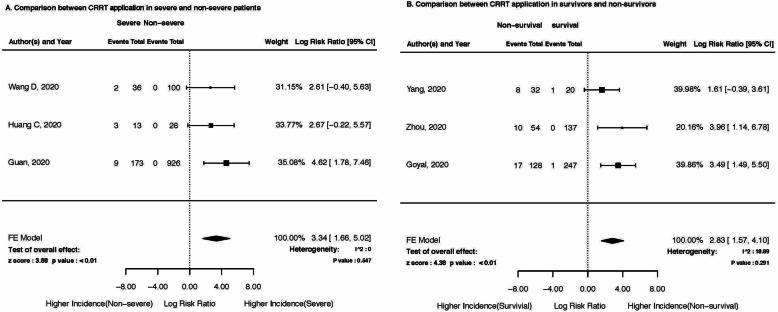
Meta-analysis of relative risk of CRRT application on severe and non-survival cases compared with non-severe and survival cases. **a** The forest plot represents the relative risk of CRRT application on severe patients compared with non-severe patients. **b** The forest plot represents the relative risk of CRRT application on non-survival cases compared with survival cases. Heterogeneity is defined based on the calculated *I*^2^ index, and fixed effect models are used to calculate the weights

Finally, CRRT was used on 5.6% (95% CI 2.6–8.6%) severe patients, 0.1% (95% CI − 0.1–0.2%) non-severe patients (Supplementary Figure 2) and 15.6% (95% CI 10.8–20.5%) non-survivors, 0.4%(95% CI − 0.2–1.0%) survivors (Supplementary Figure 3), showing the log risk ratio 3.34 (95% CI 1.66–5.02, *Z* = 3.88, *P* < 0.01) and 2.83 (95% CI 1.57–4.10, *Z* = 4.38, *P* < 0.01), respectively. (Fig. 6).

## Discussion

Our meta-analysis showed that renal impairment in patients with COVID-19 is common. The manifestations of kidney damage are diverse, ranging from abnormal urine analysis, elevated sCr or BUN levels, to AKI and renal failure for which CRRT is required. Among these renal impairments, abnormal urine analysis is the most frequent. 57.2% patients have proteinuria, in which mild proteinuria (+) as well as moderate or severe proteinuria (++ to +++) account for 38.8% and 10.6%, respectively. Cheng’s study has found that compared with patients without proteinuria, patients with proteinuria+ and ++ to +++ had a 1.5-fold and fivefold increased risk of death, respectively, indicating that proteinuria is an independent risk factor for in-hospital death of COVID-19 patients [2], so more attention should be paid to urine analysis in clinical practice.

Another main kidney damage manifestation was elevated sCr and BUN levels with incidence rate of 9.6 and 13.7%, respectively. Elevated sCr and BUN levels may be due to chronic kidney disease or AKI. Cheng’s study showed that the baseline sCr was elevated in 15.5% COVID-19 patients on admission; patients with elevated baseline of sCr were more likely to develop AKI and more severe AKI. Meanwhile, elevated baseline sCr and BUN levels was an independent risk factor for hospital mortality [2]. Therefore, we should increase more awareness to COVID-19 patients who had elevated baseline sCr.

Our meta-analysis showed that the averaged incidence of AKI was 4.5%, ranging from 1.3 to 36.4% dependent on the different severity of the illness. The exact pathogenesis of COVID-19-associated AKI is unclear. Based on the results of our meta-analysis in combination with our frontline experience, we consider that the etiology of renal impairment is likely to be diverse and multifactorial. Besides the direct attack by the SARS-CoV-2, hypoxia and hypercoagulability also contribute to kidney damage.

It has been reported that in the kidney, angiotensin-converting enzyme2 (ACE2) is highly expressed in the brush border of proximal tubular cells but not in glomerular endothelial and mesangial cells [29], which could explain the fact that the main feature of kidney injury is the damage of renal tubules. Zhong’s team in Guangzhou has also successfully isolated SARS-CoV-2 from the urine sample of an infected patient [30]. It also has been found in the kidney biopsy that SARS-CoV-2 antigens accumulated in kidney tubules in situ [31, 32]. These evidences supported the notion that SARS-CoV-2 can directly attack human kidney. In addition, COVID-19 patients exhibit obvious hypoxia symptoms and renal tubules are more susceptible to ischemia and hypoxia compared to glomeruli. Moreover, COVID-19 patients mainly showed hypercoagulant states with elevated levels of D-dimer, leading to the slow blood flow velocity in the capillaries around the renal tubules and increased risk of microthrombus formation as confirmed by autopsy results [33].

In contrast to the previous study showing that median time for the occurrence of AKI from symptom onset were 20 and 16 days in SARS and MERS patients, respectively [1], Cheng et al. have found that AKI developed 6 days after admission in most COVID-19 patients while only 2 days after admission in patients with elevated baseline of sCr [2]. Our meta-analysis also revealed that the incidence of AKI was more than fivefold higher in severe cases and non-survivors than that in non-severe cases and survivors. Since severe COVID-19 is commonly complicated with ARDS which has high demand for fluid management, optimizing fluid volume and maintaining hemodynamic stability are crucial to prevent the occurrence or progression of AKI. In addition, appropriate anticoagulant therapy might help to reduce the microthrombus formation and to alleviate renal injury.

Our meta-analysis also showed 15.6% non-survivors received CRRT, while 0.4% survivors needed CRRT, which indicated that COVID-19 patients complicated with AKI have a poor prognosis once CRRT is applied. As existing studies have not provided detailed information about survivors and non-survivors experiencing CRRT, further research is needed to determine whether early renal replacement therapy could improve the prognosis of COVID-19 patients complicated with AKI.

### Study limitations

The number of studies included was limited in terms of sample size, data availability, and methodologic quality, as most of the patients were from China. It will be better to include more studies with a broad geographic scope, to get a more comprehensive understanding of acute renal impairment in COVID-19. In addition, more detailed patient information, particularly regarding the risk of AKI and the relationship of comorbidities with AKI, was not available in most studies at the time of analyses. Another limitation of this study is that some articles have provided median and interquartile ranges but not mean and standard deviation of the sCr and BUN values. The mean and standard deviation for those data has been estimated in this study based on the median and the interquartile range, which might lead to inaccuracy to some extent.

## Conclusion

Although ARDS was the main feature of COVID-19, our meta-analysis identified the high prevalence of abnormal urine analysis and kidney dysfunction in COVID-19 patients. The overall incidence of AKI in COVID-19 patients was 3.7%, and the degree of AKI is closely associated with the severity and prognosis of COVID-19 patients.

We consider renal tubule as the main part of injury in COVID-19 patients and the etiology of renal impairment in COVID-19 patients is likely to be diverse and multifactorial. Apart from direct attack by the SARS-CoV-2, hypoxia and hypercoagulability may also contribute to the occurrence of renal injury.

Our findings indicate that it is important to increase awareness of kidney impairment in COVID-19 patients. Screening of the urine analysis, more frequent measurement of sCr, optimization of fluid volume, and appropriate anticoagulant therapy during the management of COVID-19 are essential.

## Supplementary information

**Additional file 1 : Supplementary Figure 1.** Meta-analysis of the mean value of serum creatinine and BUN in COVID-19 patients. Heterogeneity is defined based on the calculated I^2^ index, and random effect models are used to calculate the weights. The forest plots represent mean values of serum creatinine (A, B) and BUN (C, D).

**Additional file 2 : Supplementary Figure 2.** A: Forest plot of average AKI incidence in non-survivors. B: Forest plot of average AKI incidence in survivors. Heterogeneity is defined based on the I2 index calculated. A fixed effect model is used to pool the average AKI incidence in non-survivors, and a random effect model is used to pool the data of AKI incidence in survivors.

**Additional file 3 : Supplementary Figure 3.** A: Forest plot of average proportion of severe patients who required CRRT. B: Forest plot of average proportion of mild patients who required CRRT. Heterogeneity is defined based on the I2 index calculated. Fixed effect models are used to pool data of both groups.

**Additional file 4 : Supplementary Figure 4.** A: Forest plot of average proportion of non-survivors who required CRRT. B: Forest plot of average proportion of survivors who required CRRT. Heterogeneity is defined based on the I2 index calculated. Fixed effect models are used to pool data of both groups.

## Data Availability

Not applicable
