# Supplementary figures and images for: Prevalence and impact of acute renal impairment on COVID-19: a systematic review and meta-analysis
Source: Crit Care. 2020 Jun 18;24:356. doi: 10.1186/s13054-020-03065-4 (PMC7300374; doi:10.1186/s13054-020-03065-4)

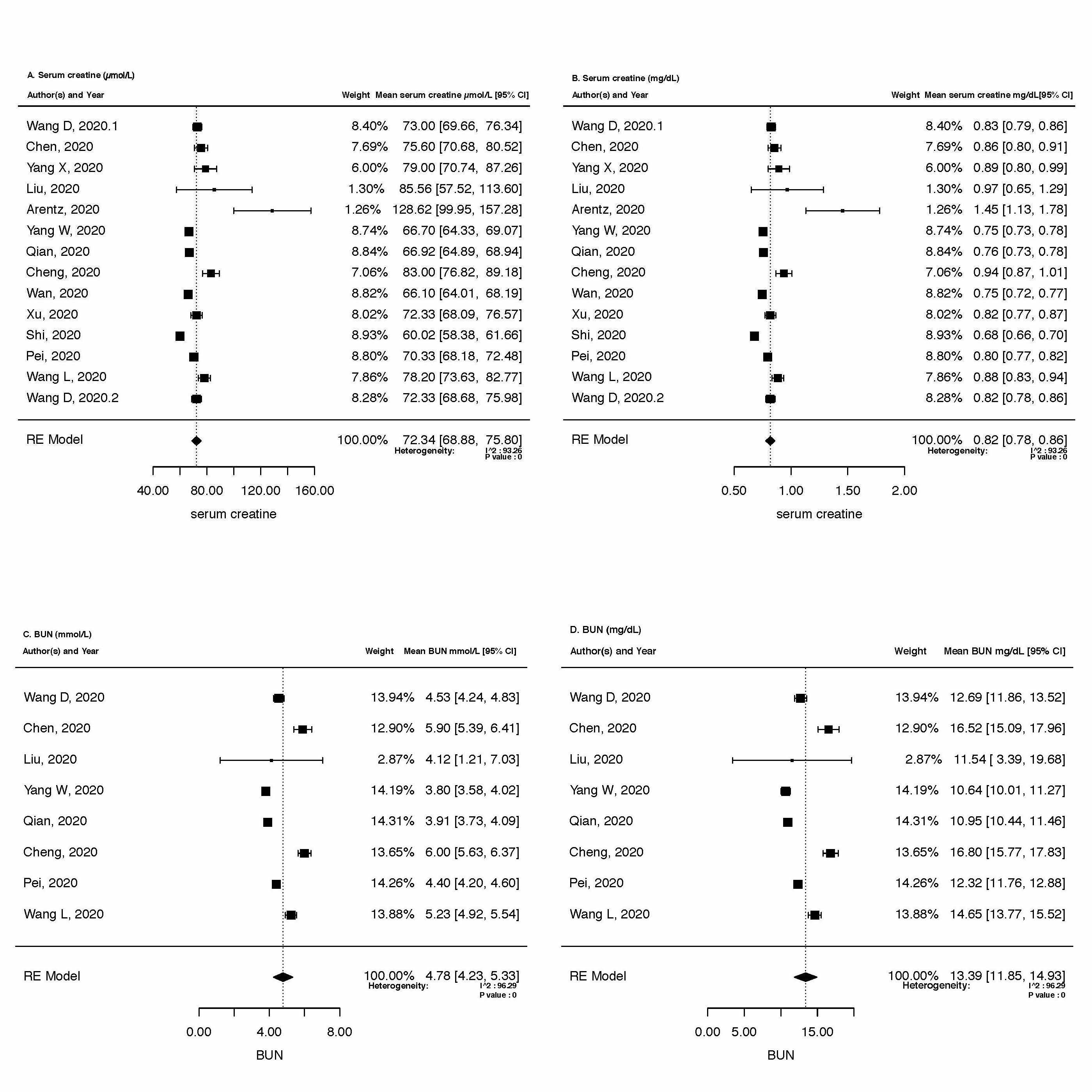

Supplement: Supplementary file 1 — Additional file 1 : Supplementary Figure 1. Meta-analysis of the mean value of serum creatinine and BUN in COVID-19 patients. Heterogeneity is defined based on the calculated I2 index, and random effect models are used to calculate the weights. The forest plots represent mean values of serum creatinine (A, B) and BUN (C, D). [file 13054_2020_3065_MOESM1_ESM.jpg]

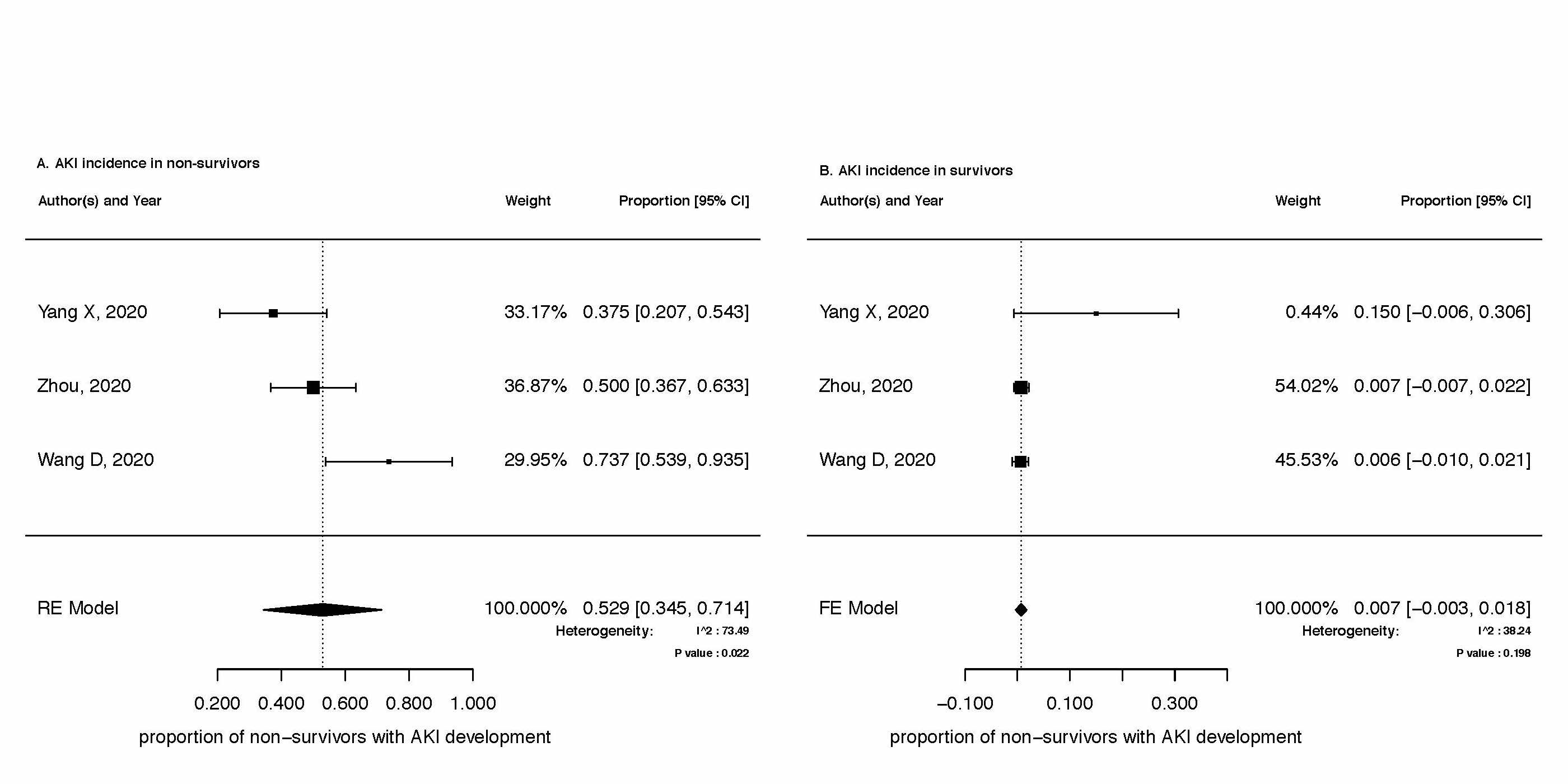

Supplement: Supplementary file 2 — Additional file 2 : Supplementary Figure 2. A: Forest plot of average AKI incidence in non-survivors. B: Forest plot of average AKI incidence in survivors. Heterogeneity is defined based on the I2 index calculated. A fixed effect model is used to pool the average AKI incidence in non-survivors, and a random effect model is used to pool the data of AKI incidence in survivors. [file 13054_2020_3065_MOESM2_ESM.jpg]

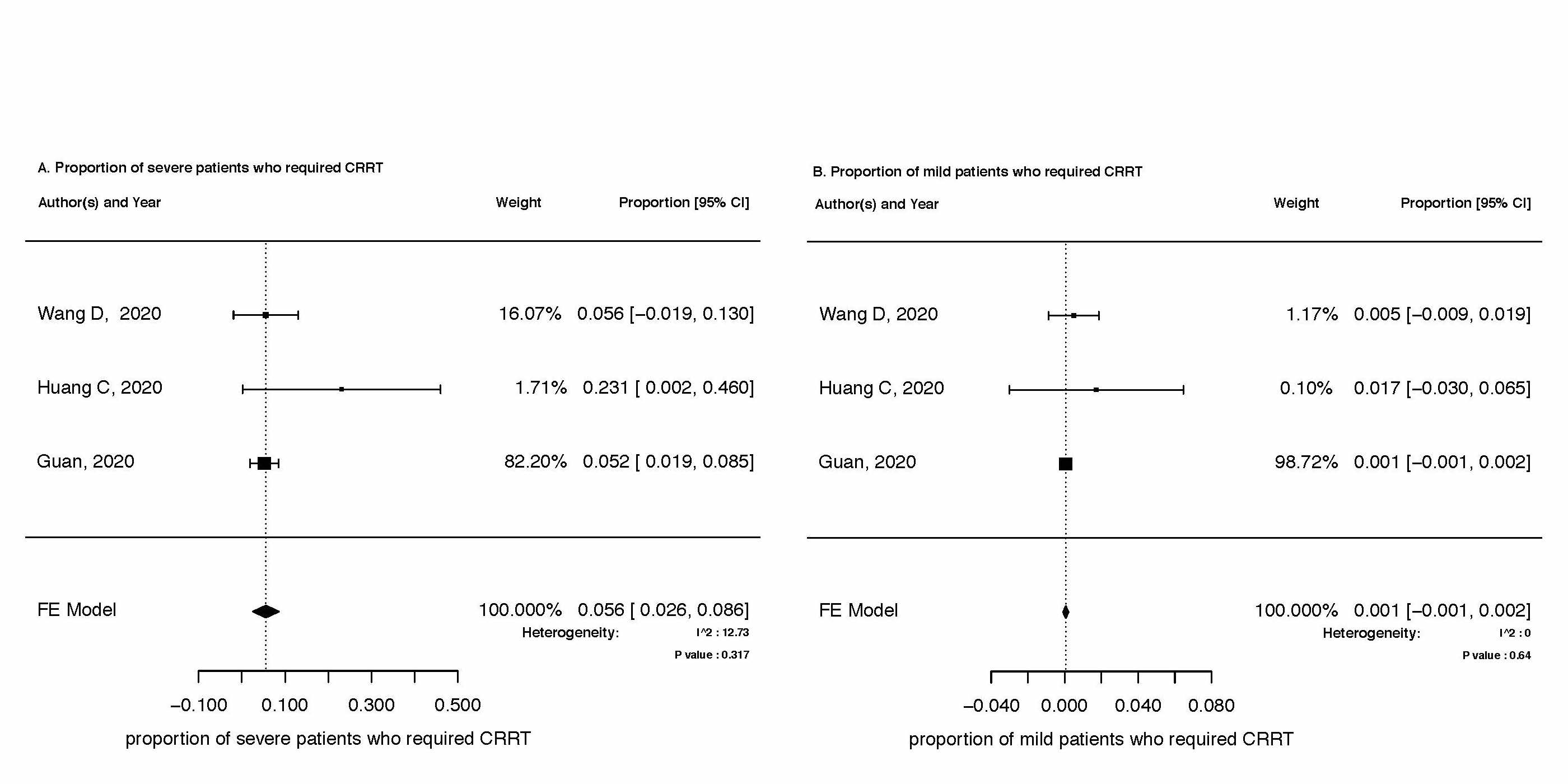

Supplement: Supplementary file 3 — Additional file 3 : Supplementary Figure 3. A: Forest plot of average proportion of severe patients who required CRRT. B: Forest plot of average proportion of mild patients who required CRRT. Heterogeneity is defined based on the I2 index calculated. Fixed effect models are used to pool data of both groups. [file 13054_2020_3065_MOESM3_ESM.jpg]

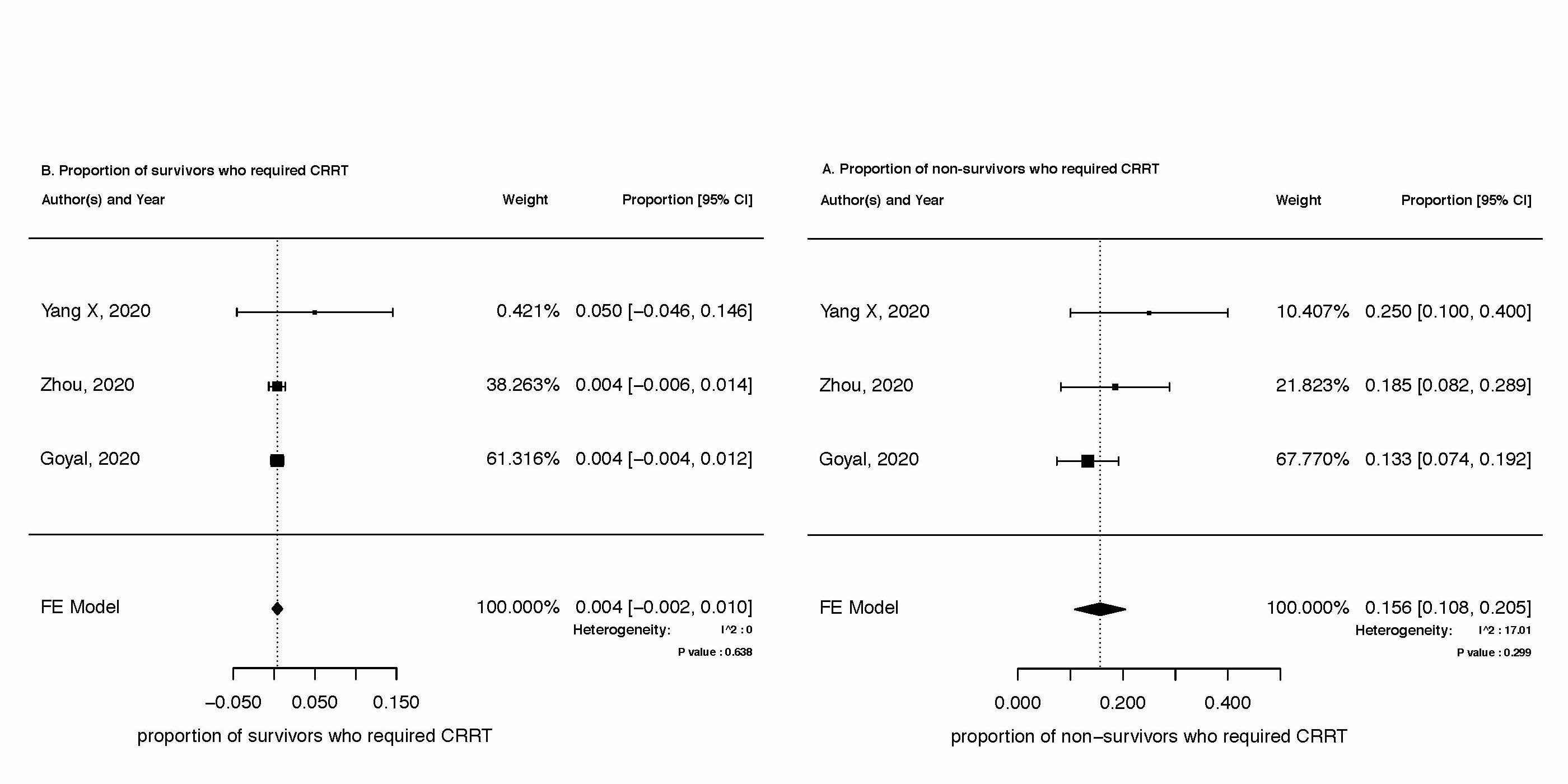

Supplement: Supplementary file 4 — Additional file 4 : Supplementary Figure 4. A: Forest plot of average proportion of non-survivors who required CRRT. B: Forest plot of average proportion of survivors who required CRRT. Heterogeneity is defined based on the I2 index calculated. Fixed effect models are used to pool data of both groups. [file 13054_2020_3065_MOESM4_ESM.jpg]
